# A Class-J Power Amplifier Implementation for Ultrasound Device Applications

**DOI:** 10.3390/s20082273

**Published:** 2020-04-16

**Authors:** Kiheum You, Seung-Hwan Kim, Hojong Choi

**Affiliations:** 1 Department of Medical IT Convergence Engineering, Kumoh National Institute of Technology, 350-27 Gumi-daero, Gumi 39253, Korea; rlgma12@kumoh.ac.kr; 2 R&D Center, Metabiomed Corporation, 215 Osongsaenmyeong1-ro, Chenongu 28161, Korea; mbclub@metabiogw.bizmeka.com

**Keywords:** class-J power amplifier, high output power, high efficiency, ultrasonic device

## Abstract

In ultrasonic systems, power amplifiers are one of the most important electronic components used to supply output voltages to ultrasonic devices. If ultrasonic devices have low sensitivity and limited maximum allowable voltages, it can be quite challenging to detect the echo signal in the ultrasonic system itself. Therefore, the class-J power amplifier, which can generate high output power with high efficiency, is proposed for such ultrasonic device applications. The class-J power amplifier developed has a power efficiency of 63.91% and a gain of 28.16 dB at 25 MHz and 13.52 dB_m_ input. The pulse-echo measurement method was used to verify the performance of the electronic components used in the ultrasonic system. The echo signal appearing with the discharged high voltage signal was measured. The amplitude of the first echo signal in the measured echo signal spectrum was 4.4 V and the total-harmonic-distortion (THD), including the fundamental signal and the second harmonic, was 22.35%. The amplitude of the second echo signal was 1.08 V, and the THD, including the fundamental signal and the second harmonic, was 12.45%. These results confirm that a class-J power amplifier can supply a very high output echo signal to an ultrasonic device.

## 1. Introduction

Ultrasound is a sound wave with a frequency of 16 kHz or higher, a frequency range above the hearing threshold of humans [1]. Ultrasound technology is used in a variety of fields, such as cleaning or fingerprint recognition in a living room, parking assistance devices in autonomous vehicles using ultrasonic distance measurement, and image-based diagnosis of muscles, tendons, and internal organs [2,3,4,5]. It is also used for the nondestructive testing and measuring of material defects and the thickness of objects [6].

In an ultrasonic system, the front-end, consists of a transceiver (transmitter + receiver) [3,7]. The transmitter is used to drive ultrasound devices by transmitting an output signal across a desired frequency range [8]. The reflected ultrasonic signal of such an ultrasound device is so small that a signal with an appropriate power amplifier output is required for accurate detection [6].

**Figure 1:**
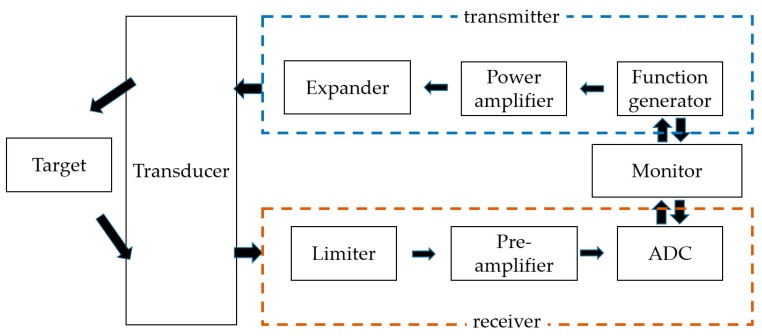
Ultrasonic system block diagram.

Figure 1 shows a simplified block diagram of the typical ultrasonic system [9]. The front-end transceiver (power amplifier, expander, preamplifier, limiter, and analog-to-digital converter (ADC)) in an ultrasonic system plays an important role in determining system sensitivity [10]. The expanders and limiters reduce unnecessary ring-down of the signal and block unwanted high voltage pulses [9]. The ADC processes the signal digitally for each image, and echo signals generated by the ultrasound device are processed and transmitted to the monitor.

Since the output and frequency characteristics of the ultrasonic transducer can be affected by the power amplifier, it is important to design the power amplifier according to the transducer characteristics [11]. Ultrasound systems require high quality images, achieved through several system attributes such as high output voltage, low noise figure, and high efficiency [12]. However, it is hard to achieve optimal power amplifier performance simultaneously [13,14,15].

Several power amplifiers have been developed for use in ultrasonic transducers. These can be divided by class models [16]. There are several features depending on the class models. The class-A power amplifiers have exceptionally high linearity but extremely low power efficiency [17]. The class-B power amplifiers have high linearity but moderate power efficiency. A class-C power amplifiers have a slightly higher power efficiency but low linearity [18]. For example, class-A and class-B power amplifiers have been suggested for general bench-top ultrasound systems to obtain high sensitivities [19]. In addition, the development of a class-C power amplifier for a point-of-care ultrasound system has been used to obtain high power efficiency [20]. The class-D power amplifiers have high efficiency with relatively low power loss and have been used to increase the high intensity power in the low frequency ultrasonic transducers [21,22,23,24]. The class-E power amplifiers provide high power efficiency and efficient switching but have extremely low linearity [21,24]. Some types of power amplifier might be suitable for low frequency transducer applications. For example, The class-D power amplifier was developed for high-power piezoelectric loads [25,26]. On the other hand, a class-E power amplifier was developed for 41.5 kHz piezoelectric ultrasonic transducers in favor of a specific frequency ranges [23,27]. Since the sensitivity of an ultrasound system generally depends on the voltage or power gain of the device, it is useful for the power amplifier of the ultrasound device to be a high-voltage or high-power amplifier [28,29]. A class-J power amplifier, boasting the advantages of high power and efficiency, have been used in telecommunication baseband systems [30,31]. Similarly, ultrasonic systems also require high output power with high efficiency, especially for miniaturized ultrasonic transducers used in the intravascular or intracardiac ultrasound systems which need to be triggered by high power signals with adequate power efficiency [6]. The maximum applied voltages of these small-size transducers are quite limited, so that effective power transfer could be more preferable to stabilize performance [32]. The previously developed class-S power amplifier has wide bandwidth to be covered for wideband ultrasonic transducer [29]. However, class-J power amplifier was designed to have high output power with high efficiency.

Therefore, we also designed the class-J power amplifiers used for superficial anatomy or intravascular applications because these frequency bands provide small depth of penetration, but good spatial resolution. Consequently, class-J power amplifiers at 25 MHz with high power and efficiency in ultrasound systems are the first to be designed for such low sensitivity ultrasound devices.

The class-J power amplifier proposed in this paper is designed to short circuit the high frequency components (of more than the third order) to satisfy the high power and high efficiency requirements collectively. A complete short circuit of the high frequency components spanning more than three orders is ideal but is affected through external matching. Therefore, a class-J power amplifier with external matching circuits was designed to improve output power efficiency.

This paper consists of the following sections. Section 2 analyzes the schematic, mathematical analysis, and simulation of a class-J power amplifier. Section 3 presents the results obtained with a class-J power amplifier. Pulse-echo experiments were further used to evaluate the ultrasonic transducers as they are a performance measurement method used to evaluate the performances of an ultrasound system or an ultrasound transducer. Finally, Section 4 presents conclusions about the research.

## 2. Materials and Methods

The proposed class-J power amplifier short-circuits the harmonic components of the third order and delivers power to the load using only the first fundamental signal and the second harmonic load. The load impedance of the second harmonic was designed so that the reactance component has a larger value than the resistance component [31,33]. Therefore, the voltage and current waveforms of the fundamental signal and the second harmonic have a phase difference of 45° and 90°, respectively. Since there is a voltage gain of the fundamental signal due to the second harmonic component being present, a power loss of cos 45° can be compensated for, and a high output is expected. In addition, the proposed amplifier was designed to have a higher output by adopting a two-stage configuration.

### 2.1. Analysis of the Class-J Power Amplifier

Class-J power amplifiers, unlike class-A and class-B power amplifiers, allow the superposition of current and voltage to increase the power transfer and efficiency capabilities [33,34]. Assuming that the drain current and voltage waveforms of a designed class-J power amplifier have only a DC component (I_DC_ and V_DC_), a fundamental frequency component (I_fundamental_ and V_fundamental_), and a second harmonic component (I_second_ and V_second_), the drain current and drain voltage are expressed as follows in Equation (1) [30,31]. The drain current and drain voltage of the first stage, total current of the first stage power amplifier (I_total(1)_ and V_total(1)_), and the second stage total current and voltage of the designed class-J power amplifier (I_total(2)_ and V_total(2)_) are expressed as follows.

(1) $$\begin{array}{ll} I_{total(1),(2)} & =I_{DC}+I_{fundamental}+I_{\mathrm{second}}\\ & =I_{DC}+I_{DC}\frac{\pi }{2}\times \sin(\theta )-I_{DC}\frac{2}{3}\times \cos(2\theta ) \end{array},$$

(2) $$\begin{array}{ll} V_{total(1),(2)} & =V_{DC}+V_{fundamental}+V_{\mathrm{second}}\\ & =V_{DC}+V_{DC}\times \alpha \times \sin(\theta )-V_{DC}\times \beta \times \cos(2\theta ) \end{array},$$

where the variables of α and β in Equation (2) represent the voltage magnitudes of the fundamental frequency load and the second harmonic load, and θ represents the conduction angle. The calculus yields two maximum and two minimum values for α and β terms, respectively. When β has a minimum value and α has a maximum value, α and β variables can be stated as √2 and ½, respectively. Therefore, the α and β variables used in Equation (2) can be represented by Equation (3).

(3) $$V_{total(1),(2)}=V_{DC}+V_{DC}\times \sqrt{2}\times \sin(\theta )-V_{DC}\times \frac{1}{2}\times \cos(2\theta ).$$

Class-J power amplifiers have a 90° phase difference with a larger reactance than the resistance of the second harmonic load to increase power [30,31,34]. To achieve the highest values α and β of the fundamental frequency load and the second harmonic voltage waveform, the fundamental frequency load needs to have a phase difference of 45°. Therefore, the second harmonic voltage and the fundamental frequency voltage are represented by Equations (4) and (5).

(4) $$V_{\mathrm{second}}=-\frac{1}{2}\times \cos(2(\theta +\frac{\pi }{4})),$$

(5) $$V_{fundamental}=\sqrt{2}\times \sin(\theta +\pi +\frac{\pi }{4}).$$

**Figure 2:**
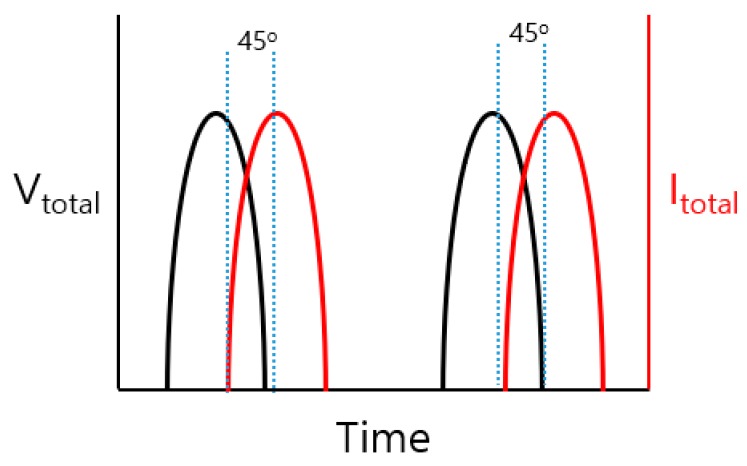
Voltage and current waveform phase differences for fundamental frequency load.

Therefore, as shown in Figure 2, the fundamental frequency voltage (V_total_) and the fundamental frequency current (I_total_) in the class-J power amplifier are 45° ahead or behind depending on whether the reactance component of the second harmonic load is a capacitance component or an inductance component.

As a result, the voltage gain of the fundamental frequency load exists due to the second harmonic component, so the power loss of cos 45° can be compensated, which is useful to yield high output power.

Normalizing the output voltage and output current (I_DC_ = I_peak_/π) flowing through the load can be represented by the Equations (6) and (7) [30,31]. The currents and voltages at the first and second stages I_total(1),(2)_ and V_total(1),(2)_ are as follows.

(6) $$I_{total(1),(2)}=\frac{I_{peak}}{2}\times \sin(\theta )-\frac{2\times I_{peak}}{3\pi }\cos(2\theta ),$$

(7) $$V_{total(1),(2)}=\sqrt{2}\times V_{DC}\times \sin(\theta +\pi +\frac{\pi }{4})-\frac{1}{2}\times \cos(2(\theta +\frac{\pi }{4})).$$

The DC power consumption (P_DC_) of the two-stage class-J power amplifier can be expressed by multiplying the DC voltage (V_DC(1)_ and V_DC(2)_) with DC current (I_DC(1)_ and I_DC(2)_) as shown in Equation (8). Using Equations (1) and (3), the DC power equation of the designed class-J power amplifier can be represented as follows by in Equation (8).

(8) $$P_{DC}=V_{DC(1)}\times I_{DC(1)}+V_{DC(2)}\times I_{DC(2)}=V_{DC(1)}\times \frac{I_{peak(1)}}{\pi }+V_{DC(2)}\times \frac{I_{peak(2)}}{\pi },$$

P_DC_ can be expressed as follows using Equations (6) and (7). P_DC_ value depends on the total voltages and currents with their phase differences [30,31].

(9) $$\begin{array}{ll} P_{DC} & =\frac{V_{total(1)}}{\sqrt{2}\sin(\theta +\pi +\frac{\pi }{4})-\frac{1}{2}cos(2(\theta +\frac{\pi }{4}))}\times \frac{I_{total(1)}}{\frac{\pi }{2}\sin(\theta )-\frac{2}{3}\cos(2\theta )}\\ & +\frac{V_{total(2)}}{\sqrt{2}\sin(\theta +\pi +\frac{\pi }{4})-\frac{1}{2}cos(2(\theta +\frac{\pi }{4}))}\times \frac{I_{total(2)}}{\frac{\pi }{2}\sin(\theta )-\frac{2}{3}\cos(2\theta )} \end{array}.$$

The output power (P_OUT_) of the class-J power amplifier can be expressed by multiplying the DC voltage (V_DC(1)_ and V_DC(2)_) with output current (I_peak(1)_ and I_peak(2)_) as shown in Equation (10). Using Equations (1) and (3), the output power equation of the designed class-J power amplifier can be represented as follows by in Equation (10).

(10) $$P_{OUT}=\frac{1}{2}(\frac{I_{peak(1)}}{2}\times V_{DC(1)}\times \sqrt{2}\times \cos(\frac{\pi }{4}))+\frac{1}{2}(\frac{I_{peak(2)}}{2}\times V_{DC(2)}\times \sqrt{2}\times \cos(\frac{\pi }{4})),$$

P_OUT_ can be expressed as follows through Equations (6) and (7). P_OUT_ value depends on the total voltages and currents with their phase differences [30,31].

(11) $$\begin{array}{ll} P_{OUT} & =\frac{1}{16}\times \{\frac{V_{total(1)}\times \sqrt{2}\cos(\frac{\pi }{4})}{\sqrt{2}\sin(\theta +\pi +\frac{\pi }{4})-\frac{1}{2}cos(2(\theta +\frac{\pi }{4}))}\times \frac{I_{total(1)}\times \pi }{\frac{\pi }{2}\sin(\theta )-\frac{2}{3}\cos(2\theta )}\\ & +\frac{V_{total(2)}\times \sqrt{2}\cos(\frac{\pi }{4})}{\sqrt{2}\sin(\theta +\pi +\frac{\pi }{4})-\frac{1}{2}cos(2(\theta +\frac{\pi }{4}))}\times \frac{I_{total(2)}\times \pi }{\frac{\pi }{2}\sin(\theta )-\frac{2}{3}\cos(2\theta )}\} \end{array}.$$

Efficiency (η) of the two-stage class-J power amplifier can be found as output power divided by DC power. Through Equations (9) and (11), the efficiency is expressed as follows [35].

(12) $$\eta =\frac{P_{OUT}}{P_{DC}},$$

The power added efficiency (PAE) of two-stage class-J power amplifier can be obtained by subtracting the input power (P_IN_) from the output power (P_OUT_) and dividing the result by the DC power consumption (P_DC_) [36]. Through Equations (9) and (11), the PAE of the designed class-J power amplifier can be derived as shown in Equation (13) [37].

(13) $$PAE=\frac{P_{OUT}-P_{IN}}{P_{DC}}\times 100\%.$$

### 2.2. Schematic of the Class-J Power Amplifier

**Figure 3:**
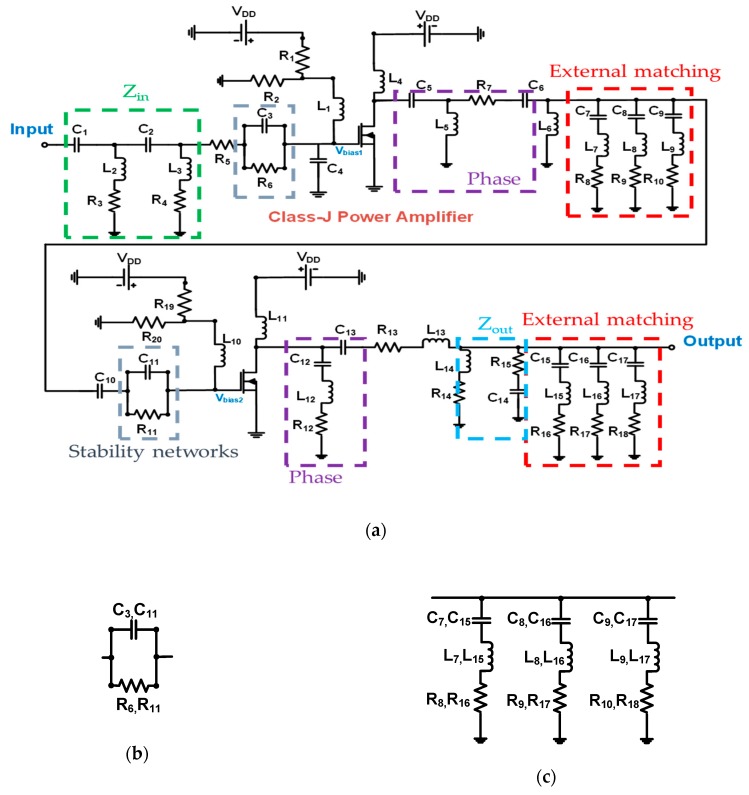
(**a**) Schematic diagram of a class-J power amplifier with a resistor divider; (**b**) the stability networks; (**c**) external matching–component short circuit above third harmonics.

Figure 3a is a schematic of a class-J power amplifier design. A bias voltage of 3.5 V was applied to the gates to be amplified in a two-stage class-J power amplifier. The gate and drain inductors (L_1_, L_4_, L_10_, and L_11_) applying the DC voltage use a choke inductor to minimize the DC voltage drop. As shown in Z_in_ (C_1_, C_2_, L_2_, L_3_, R_3_, and R_4_) and Z_out_ (L_14_, C_14_, R_14_, and R_15_) of Figure 3a, 50 Ω impedance matching was performed to match the center frequency at the input and second stage of the first stage together with the components of the power amplifier. In the phase part of the first stage and the second stage of Figure 3a, the phase matching circuits (C_5_, C_6_, L_5_, L_6_, R_7_, C_12_, L_12_, R_12_, and R_13_) were constructed to generate each phase difference between the second harmonic signal and the fundamental frequency components that are characteristic of the class-J power amplifier. In addition, an electrolytic capacitor (220 μF) and three other capacitors (0.1 μF, 1000 pF, and 47 pF) were used between the DC power supply and the resistor to reduce the noise signal of the DC power supply. As shown in Figure 3b, the stability network through the R–C device (C_3_ and C_11_, and R_6_ and R_11_) is formed in the gate part of each transistor to form a frequency band stabilization role. Therefore, high gain could be achieved.

The inductive-resistive matching circuit was not used because it may not be suitable for certain high-frequency transducer which generate long ring-down in the echo signals. The reason is that ring down affects axial resolution in certain high-frequency transducer operation. Several matching circuit structures were also used using the series capacitor with shunt inductor or series inductor with shunt capacitor [38]. Conventional class-J power amplifiers have shorted more than the third harmonics through the internal capacitors of transistors operating in the GHz band [31,33,34]. The class-J power amplifier in this paper shorted the frequency through external matching for operation in the MHz band because of the ultrasonic transducer frequency ranges. As shown in Figure 3c, the R-L-C device (C_7_, C_15_, L_7_, L _15_, R_8_, R _16_, C_8_, C _16_, L_8_, L _16_, R_9_, R _17_, C_9_, C _17_, L_9_, L _17_, R_10_, and R _18_) was connected to short the third harmonic component. Therefore, the external matching network reduces harmonic distortions, generating high efficiency. Table 1 lists the device values for the designed class-J power amplifier.

**Figure 4:**
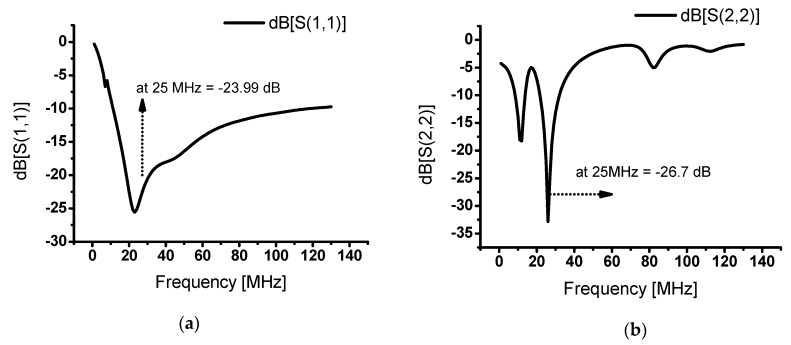

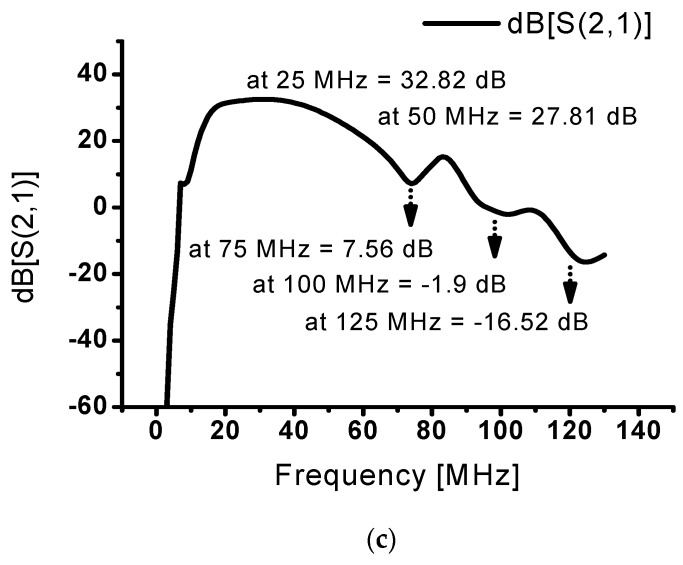
S-parameter simulation data of designed class-J power amplifier. (**a**) S(1,1), (**b**) S(2,2), and (**c**) S(2,1).

Figure 4 shows the S-parameter simulation data of the designed class-J power amplifier. The S-parameter values such as S(1,1) or S(2,2) less than −10 dB at a specific frequency suggests well matched input or output ports [31,39,40]. The S(1,1) and S(2,2) parameters are input and output reflection coefficient S-parameters [41,42]. These data indicate whether the input and output at the input and output ports are well matched for a specific frequency band [43]. Figure 4a shows the input reflection coefficient S(1,1), which was designed as −23.99 dB at 25 MHz. Figure 4b shows the output reflection coefficient S(2,2), which was designed as −26.71 dB. The S(2,1) is related to the power gain of the power amplifier [44]. Figure 4c shows the transfer coefficient S(2,1), which was designed to be 32.82 dB at 25 MHz. Therefore, this simulation data confirmed that the best performances were achieved at 25 MHz.

**Figure 5:**
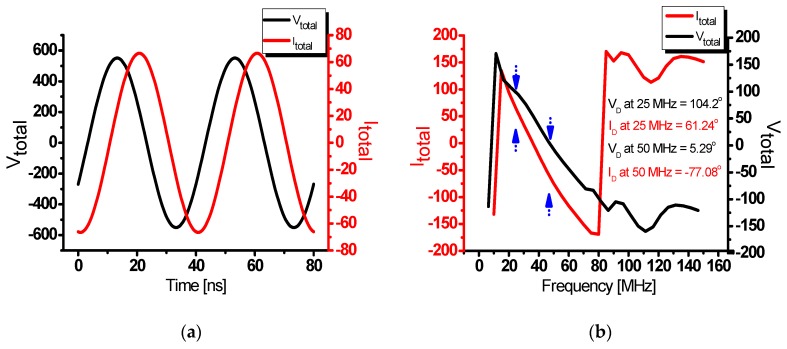
(**a**) Simulated drain voltage (V_total_) and current (I_total_) waveforms over time and (**b**) Simulated voltage (V_total_) and current (I_total_) phase differences over frequency.

Figure 5a shows the voltage (V_total_) and current (I_total_) waveforms of the drain over time. The voltage and current waveforms with phase difference were measured. Figure 5b shows the phase differences over the frequency band. A phase difference of approximately 43° was shown in the 25 MHz fundamental frequency, and a phase difference of approximately 82.4° was measured in the 50 MHz second harmonic frequency. The phase difference of the fundamental frequency load of an ideal class-J power amplifier is 45° [30,31,33]. The phase difference of the second harmonic load is 90°, so it was designed to be close to these values.

The simulated results of the power amplifiers are different to the measured results because the power amplifier simulation library cannot accurately contain temperature variances of the components and external environment [30,45,46]. Even so, the measured current data was also not accurate so we measured the PAE and the gain of the power amplifier [43,47]. The ultrasonic devices are nonlinear devices with respect to different frequencies and voltages [48]. Therefore, the power amplifier performances need to be measured to be applied to ultrasonic transducers. In the next chapter, we will show the measured performance results of the power amplifiers using pulse-echo responses.

## 3. Results

**Figure 6:**
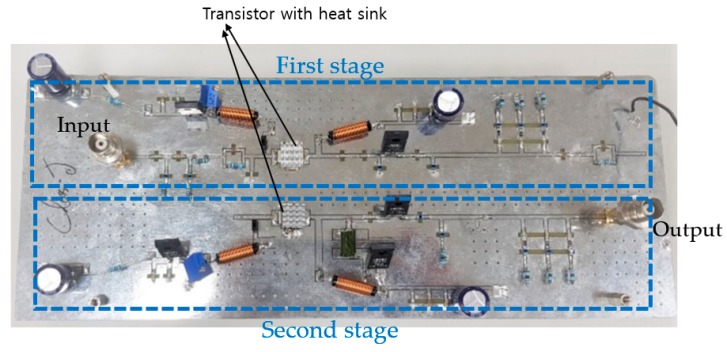
Printed circuit board (PCB) of a class-J power amplifier.

Figure 6 shows the printed circuit board (PCB) of the class-J power amplifier composed of two-stage. Power resistors, high-power choke inductors, and electronic capacitors were used to function fully in high voltage environments.

### 3.1. Performance Analysis

**Figure 7:**
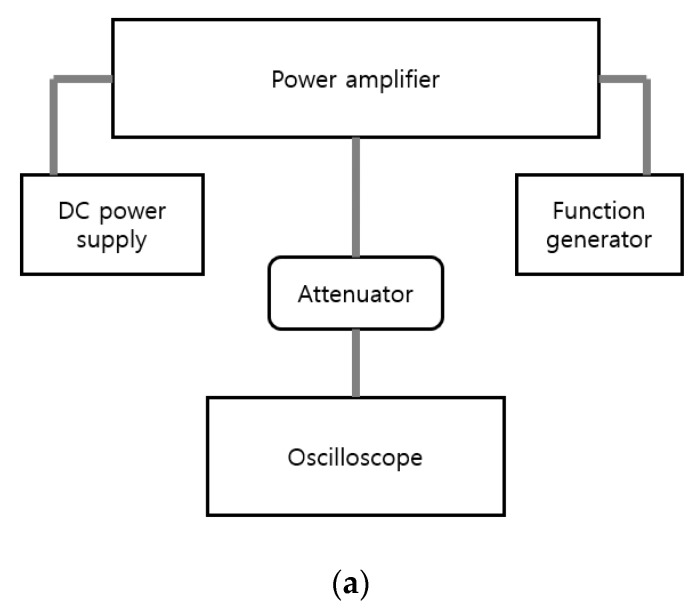

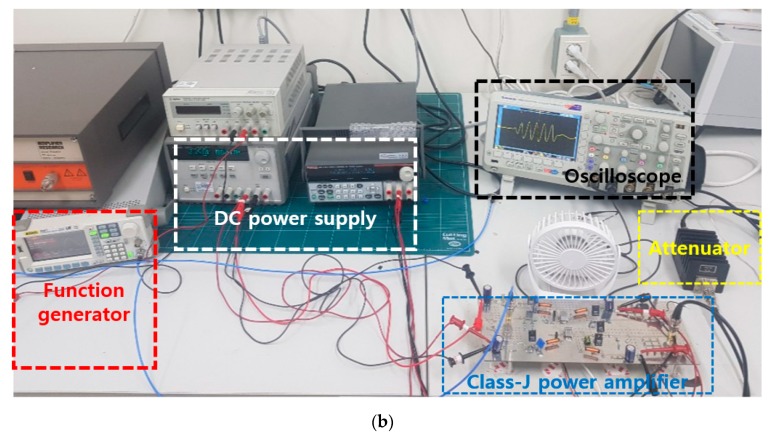
(**a**) Block diagram showing how to measure the performance of a class-J power amplifier, (**b**) the measurement environment.

**Figure 8:**
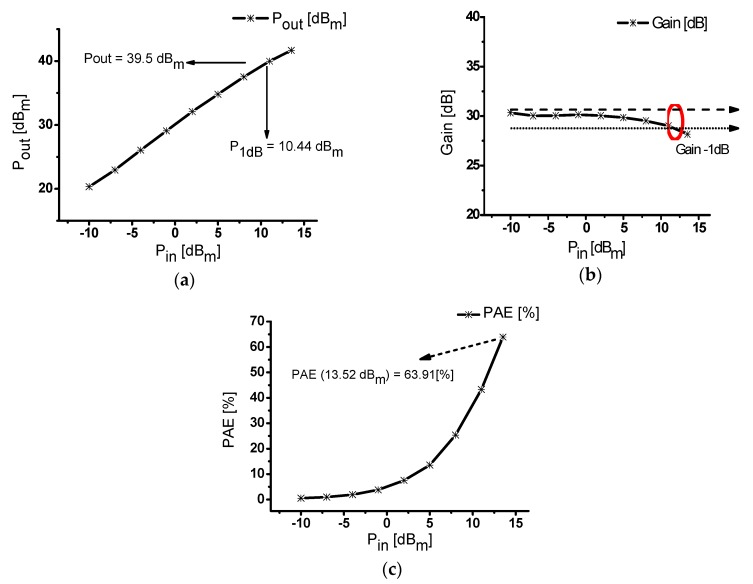
(**a**) Input power versus output power; (**b**) input power versus power gain; (**c**) input power versus PAE observed in a class-J power amplifier with an input frequency of 25 MHz.

Figure 7a,b shows the experimental evaluation method used to measure the power gain and power efficiency of the class-J power amplifier itself. In the function generator, a 5-cycle sinewave was generated with respect to the operating frequency or amplitude of the input voltage. The pulse repetition interval (PRI) is 10 ms. To prevent damage due to overvoltage of the oscilloscope, we used a 100 W, 40 dB attenuator. External coolers and heat sinks were also used to obtain accurate measurement data. In Figure 8, the power gain, power efficiency, and output voltage were measured as a function of input voltage and frequency.

Figure 8a shows the performance measurement when the input power of a class-J power amplifier was in the range of −10 to 13.5 dB_m_ at 25 MHz. At an input of 13.5 dBm, the DC current was 980 mA and the output was 41.69 dB_m_. In addition, the 1-dB compression point (P_1dB_) of the designed power amplifier was measured. P_1dB_ represents the maximum power point available before reaching the saturation power of the power amplifier, the output power at the point where the power gain of the power amplifier was reduced by −1 dB [37]. P_1dB_ of the designed power amplifier was measured with an output power of 39.5 dB_m_ at the input 10.44 dB_m_ where the gain was reduced by −1 dB. In addition, when the input was 13.52 dB_m_, the output was 41.69 dB_m_. Figure 8b shows the performance measurements when the input power of a class-J power amplifier was in the range of −10 to 13.5 dB_m_ at 25 MHz. At an input of 13.5 dB_m_ the gain was measured at 28.17 dB. The reduced point of −1 dB was 29.45 dB, and the P_1dB_ was 10.44 dB_m_. Figure 8c shows the power gain versus frequency at the input of 3 V_P-P_. The PAE of 63.91% was measured at an input of 13.52 dB_m_. The PAE also increased with input increments, which shows that the designed power amplifier has both high power and high efficiency. Table 2 summarizes the measured output power, power gain, and the PAE versus input power of the designed class-J power amplifier.

**Figure 9:**
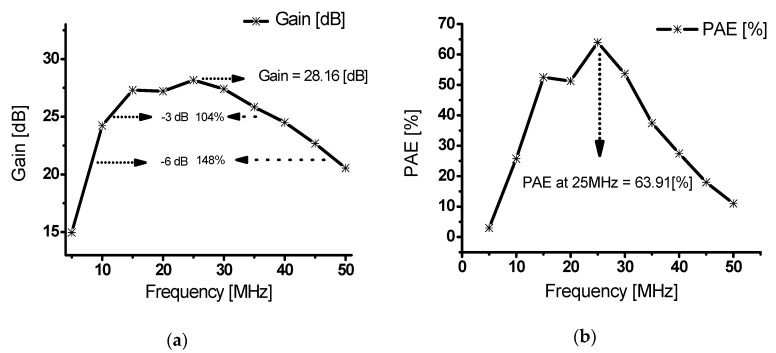
(**a**) Output power of a class-J power amplifier versus frequency with an input voltage of 3 V_P-P_, (**b**) PAE of a class-J power amplifier versus frequency with an input voltage of 3 V_P-P_.

Figure 9a shows the power gain versus frequency at an input of 3 V_P-P_. The gain was measured at 28.16 dB at the center frequency of 25 MHz. In addition, the bandwidth at −6 dB was 148% and the bandwidth at −3 dB was 104%. Figure 9b shows the PAE versus frequency at an input of 3 V_P-P_. At the center frequency of 25 MHz, the PAE was highest, at 63.91%. Table 3 shows the input frequency, measured output power, measured power gain and PAE.

### 3.2. Pulse-Echo Analysis

**Figure 10:**
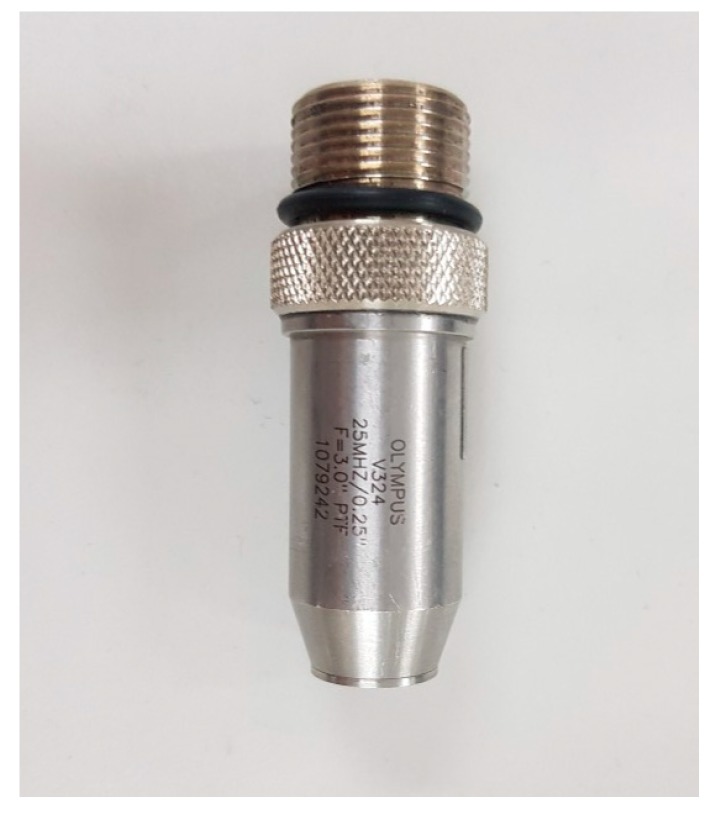
Twenty-five megahertz ultrasonic transducer.

Pulse-echo experiments are a basic indicator of the performance testing ultrasonic components or systems, including ultrasonic transducers [49,50,51]. Ultrasonic waves were generated by applying an electrical signal to a transducer with a piezoelectric effect [52]. The electrical signal from the transducer was detected based on the wave reflected from the target. In this paper, ultrasonic experiments and measurements were performed using a 25 MHz transducer as shown in Figure 10. A single element transducer provided by Olympus (Shinjuku-ku, Tokyo, Japan) was employed for the pulse-echo test.

**Figure 11:**
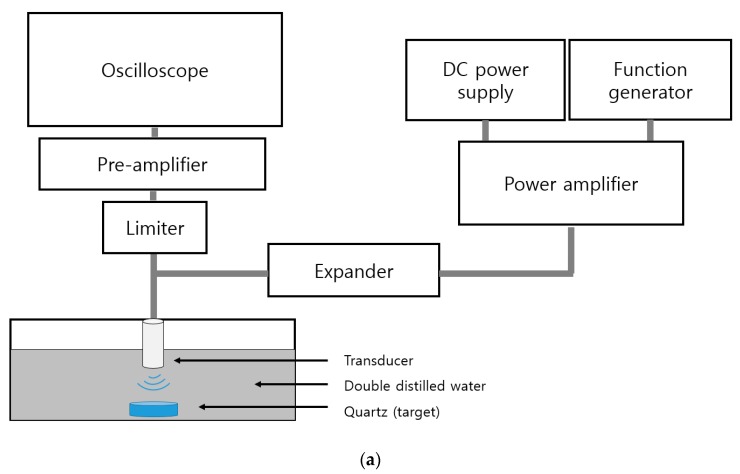

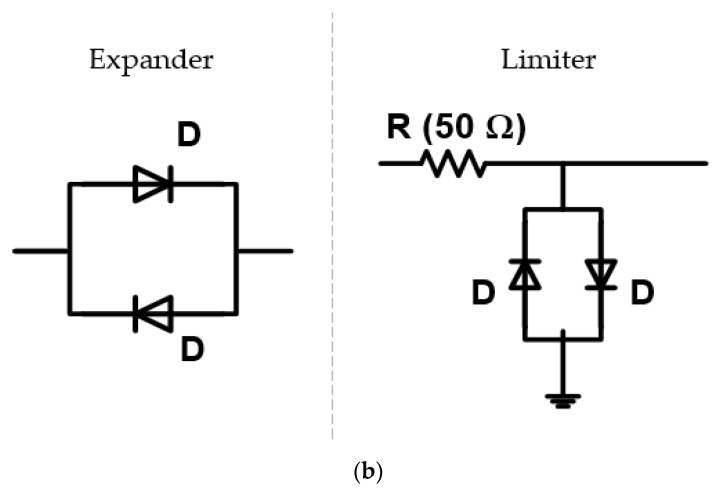
(**a**) Pulse-echo signal measurement setup for a class-J power amplifier; (**b**) Expander and limiter circuit model.

As shown in Figure 11a, a DC voltage was applied to the gate and drain through the DC power supply, and the frequency and input voltage were changed through the function generator. The water tank was 80% filled with double distilled water, which is similar to the blood in a person’s body [53,54,55]. The signal was amplified by the power amplifier and 100% reflected by the quartz target through the transducer in the water tank. The reflected ultrasonic signal was amplified by the pre-amplifier and measured by the oscilloscope. Figure 11b shows the circuit model of the expander and limiter. The limiter was connected in parallel with a pair of diodes and a resistor to protect the oscilloscope and pre-amplifier by removing high voltage signals [56]. The expanders had a pair of diodes connected in series to reduce the ring-down of the signal [9,57].

**Figure 12:**
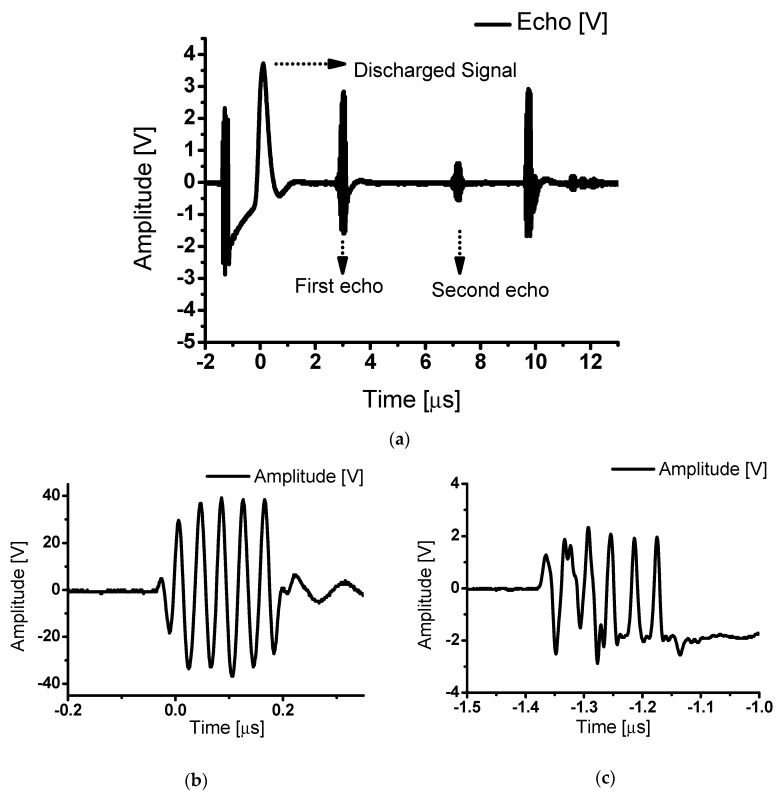
(**a**) Pulse-echo experiment of class-J power amplifier; (**b**) the output of the class-J power amplifier and expander; (**c**) the received discharged signal of the limiter after the transducer.

Figure 12a shows the waveforms of pulse-echo measurements with a class-J power amplifier using an ultrasonic transducer. Discharged signals that passed through the pre-amplifier were detected, and the first and second echo signals reflected by the target (quartz) were measured using the ultrasonic transducer. The target (quartz) reflected 100% of the ultrasonic signal [58,59]. Figure 12b shows the output signal of the class-J power amplifier and its expander circuit. In addition, Figure 12c shows the limiter after the ultrasonic transducer. The waveform of the class-J power amplifiers was passed through expanders and limiters which have non-linear components such as diodes. Therefore, some distorted waveforms must be shown in Figure 12c. Additionally, the harmonic signal through the fast Fourier transform (FFT) was analyzed to find the total harmonic distortion (THD). The THD represents the distortion caused by unnecessary harmonic components in a signal. The THD can be calculated using Equations (14)–(16) [60,61,62].

(14) $$THD=\frac{\sqrt{\mathrm{Second}.Harmonic^{2}+Third.Harmonic^{2}}}{Fundamental},$$

(15) THD (dB)=20⋅logTHD,

(16) THD (%)=100⋅THD.

**Figure 13:**
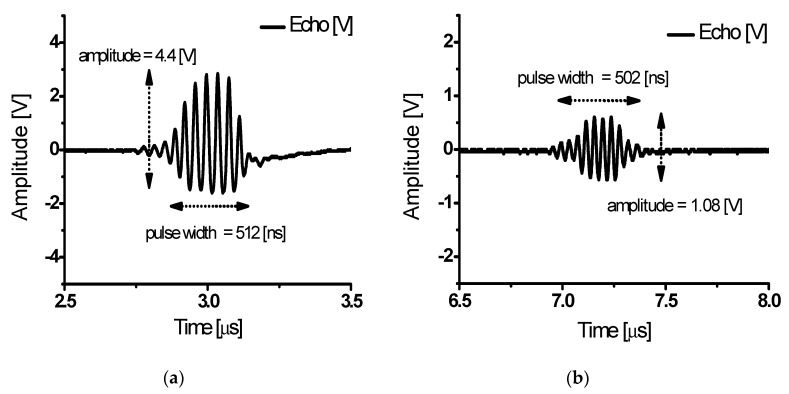
(**a**) First echo signals from a class-J power amplifier with an input voltage of 3 V_P-P_ and input frequency of 25 MHz; (**b**) second echo signals from a class-J power amplifier with an input voltage of 3 V_P-P_ and input frequency of 25 MHz.

Class-J power amplifiers boast a high output power by delivering the load as a component of the fundamental and second harmonics. Figure 13a shows the first echo signal with 4.4 V amplitude and 512 ns pulse width. Figure 13b shows a second echo signal with 1.08 V amplitude and 512 ns pulse width. Figure 13a,b show that the echo signals have very high outputs.

**Figure 14:**
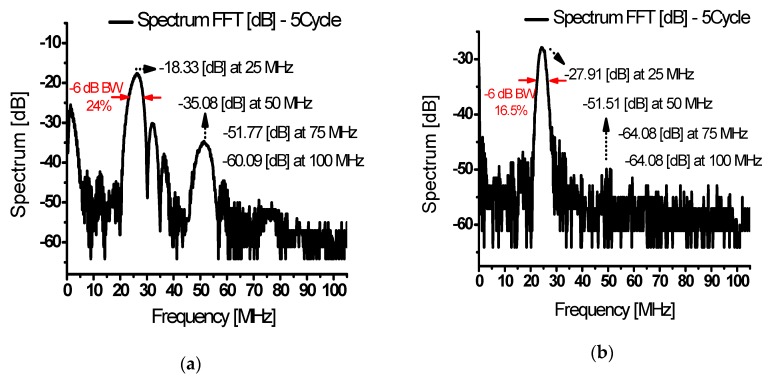
(**a**) FFT data of the first echo signals and (**b**) FFT data of the second signals from a class-J power amplifier with an input voltage of 3 V_P-P_ and input frequency of 25 MHz.

Figure 14a shows the FFT for the first echo signal as shown in Figure 13a. The fundamental, second, third, and fourth harmonic components were measured as −18.33 dB at 25 MHz, −35.08 dB at 50 MHz, −51.77 dB at 75 MHz, and −60.09 dB at 100 MHz. With the fundamental signal and second harmonics, the harmonic content above the 3rd order was significantly reduced. After converting the dB value into voltage, the calculated THD was −13.02 dB (22.35%). Figure 14b shows the FFT for the second echo signal as shown in Figure 13b. The fundamental, second, third, and fourth harmonic components were measured as −27.91 dB at 25 MHz, −51.51 dB at 50 MHz, −64.08 dB at 75 MHz, and −64.08 dB at 100 MHz. The calculated THD was −18.1 dB (12.45%). Comparing Figure 14a,b, Figure 14a has a fundamental signal and second harmonic with high output but a slightly high THD. Figure 14b shows a reduced fundamental signal and several harmonics (relatively reduced), with the advantage of a small output with low signal distortion. According to the result in Figure 14a, the echo signals have 24% and 16.5% of the −6 dB bandwidth at 25 MHz and −6 dB bandwidth of the designed class-J power amplifier is 148% in Figure 9 which can sufficiently be covered.

Table 4 shows the performance comparison between the developed class-C, D, and E power amplifiers and the class-J power amplifier proposed in the paper. These are used for different conditions of operating frequency, input voltage, output power, and for different transducer application types.

## 4. Conclusions

Class-J power amplifiers have been used in RF or telecommunication systems due to the advantages of their high power with high efficiency. Miniaturized ultrasonic transducers also require high power with limited maximum allowable voltages, thus a class-J power amplifier with external matching circuit was designed to be used for such ultrasonic devices with low sensitivities.

Class-J power amplifiers deliver load power in terms of a fundamental signal and second harmonic components. In the fundamental signal, the drain voltage and current waveforms have 45° of phase difference and the second signal has 90° of phase difference, making it suitable for use in an ultrasound transducer in the MHz range because the power amplifier’s high efficiency and output can trigger such an ultrasonic device. The designed class-J power amplifiers are suitable for high frequency ultrasound systems that require the use of relatively low sensitivity ultrasound transducers, or for intravascular ultrasound systems with miniaturized ultrasound transducers that require high output and low heat.

To design a class-J power amplifier, external matching was added to the short-circuit components above the third harmonic, and enhanced output and gain values were obtained. When 3 V_P-P_ was applied at 25 MHz, the output power was 41.69 dB_m_, the gain 28.16 dB, and the PAE 63.91%. In the pulse-echo experiment, there was a difference between the first echo signal and the second echo signal. In the first echo, the signals with fundamental signals and harmonic components were measured at 4.4 V with a THD of 22.35%. The second echo signals with fundamental signals and harmonic components were measured at 1.08 V amplitude and 12.45% THD.

As a result, the class-J power amplifiers have both high output and efficiency, capable of generating a very high echo signal output. Consequently, our designed class-J power amplifiers would be beneficial for such ultrasonic transducer applications.

## Figures and Tables

**Table 1:** Numerical values of the circuit elements depicted in Figure 3.

Component	Description	Component	Description
C_1_	390 pF	L_1_, L_4_, L_10_, L_11_	1 μH
C_2_, C_3_, C_10_, C_11_	1000 pF	L_2_, L_3_, L_6_, L_13_, L_14_	470 nH
C_4_	27 pF	L_5_, L_8_	68 nH
C_5_	1500 pF	L_7_	100 nH
C_6_	270 pF	L_9_, L_17_	39 nH
C_7_, C_15_	39 pF	L_12_	390 nH
C_8_, C_9_	41 pF	L_15_	120 nH
C_12_	820 pF	L_16_	56 nH
C_13_	120 pF	R_1_	650 Ω
C_14_	750 pF	R_2_	180 Ω
C_16_, C_17_	43 pF	R_3_, R_4_	110 Ω
R_8_, R_9_, R_10_	3 Ω	R_5_	120 Ω
R_16_, R_17_, R_18_	3 Ω	R_6_, R_11_	100 Ω
R_12_, R_14_	10 Ω	R_7_	30 Ω
R_13_	4 Ω	R_15_	80 Ω
R_19_	2 kΩ	R_20_	360 Ω

**Table 2:** Output power, gain, and power added efficiency of the designed class-J power amplifier performance, corresponding to different inputs.

Pin [dB_m_]	Pout [dB_m_]	Gain [dB]	PAE [%]
−10	20.34	30.34	0.51
−7	22.95	30.03	0.93
−4	26.06	30.04	1.88
−1	29.08	30.14	3.78
2	32.08	30.04	7.53
5	34.81	29.85	13.54
8	37.53	29.52	25.34
11	39.99	29.01	43.29
13.52	41.69	28.17	63.91

**Table 3:** Output power, gain, and power added efficiency of the designed class-J power amplifier performance, corresponding to frequencies.

Frequency [MHz]	Output [V_P-P_]	Gain [dB]	PAE [%]
5	16.8	14.96	2.97
10	48.8	24.23	25.76
15	69.6	27.31	52.49
20	68.8	27.21	51.27
25	76.8	28.16	63.91
30	70.4	27.41	53.70
35	58.8	25.85	37.44
40	50.4	24.51	27.48
45	40.8	22.67	17.97
50	32.0	20.56	11.02

**Table 4:** Summarized performances of class-C power amplifier [20], class-D power amplifier [26], class-E power amplifier [23], and the developed class-J power amplifier.

Parameters	Class-C	Class-D	Class-E	Our Work
Input voltage	5 [V_P-P_]	2.5 [V_P-P_]	5 [V_P-P_]	3 [V_P-P_]
Output power	30 [dB_m_]	39.54 [dB_m_]	29.03 [dB_m_]	41.69 [dB_m_]
Frequency	25 [MHz]	41 [KHz]	1010 [KHz]	25 [MHz]
PAE	–	–	–	63.91[%]
Drain efficiency	–	–	90[%]	–
Application	Piezoelectric transducer	Langevin transducer	MRI-compatible Piezoelectric transducer	Piezoelectric transducer
